# NeonaTal Assisted TelerehAbilitation (T.A.T.A. Web App) for Hearing-Impaired Children: A Family-Centered Care Model for Early Intervention in Congenital Hearing Loss

**DOI:** 10.3390/audiolres12020021

**Published:** 2022-03-28

**Authors:** Emma Landolfi, Grazia Isabella Continisio, Valeria Del Vecchio, Nicola Serra, Ernesto Burattini, Massimiliano Conson, Elio Marciano, Carla Laria, Annamaria Franzè, Antonio Caso, Anna Rita Fetoni, Rita Malesci

**Affiliations:** 1 Unit of Audiology, Department of Neuroscience, Reproductive Sciences and Dentistry, University of Naples Federico II, via Pansini 5, 80131 Naples, Italy; emyla@hotmail.com (E.L.); ernesto.burattini@unina.it (E.B.); marciano@unina.it (E.M.); carla.laria@unina.it (C.L.); annamaria.franze@unina.it (A.F.); antocaso@gmail.com (A.C.); annarita.fetoni@unina.it (A.R.F.); rita.malesci@unina.it (R.M.); 2 Department of Public Health, University Federico II of Naples, via Pansini 5, 80131 Naples, Italy; nicola.serra5@gmail.com; 3 Department of Psychology, University of Campania Luigi Vanvitelli, viale Ellittico 31, 81100 Caserta, Italy; massimiliano.conson@unicampania.it

**Keywords:** permanent hearing impairment, early hearing detection and intervention program, telemedicine, habilitation

## Abstract

Background: An early hearing detection and intervention program (EHDI) is the first step for the habilitation of children with permanent hearing impairment (PHI). Actually, early intervention programs have increasingly shifted toward family involvement, emphasizing that the child’s family should take an active role in the habilitation process. Therefore, familiar empowerment is the best way to improve a child’s emerging abilities. The aim of this study was to investigate parental self-efficacy beliefs and involvement as well as the language skills of deaf or hard of hearing DHH children who were habilitated with hearing aids and followed using the T.A.T.A web app (NeonaTal Assisted TelerehAbilitation), an example of asynchronous telepractice. Methods: The study describes the early stages of the habilitation program of 15 PHI children followed through the T.A.T.A. web app, which empowers families through a weekly questionnaire submitted during the first 270 to 360 days of their child’s life, for 14 weeks. The family involvement rate scale (FIRS) was used to evaluate parental compliance, and all children received in-person visits at the beginning and at the end of the training period. Results: The children showed greater auditory perceptual skills at the end of the training period on the basis of both the Infant Listening Progress Profile (ILiP) score and the Categories of Auditory Performance (CAP) and FIRS scales. In other words, the auditory skills improved with age as well as with parental participation. Conclusions: The T.A.T.A. web app promotes a proactive management and a tailored habilitation through an active familiar involvement, easily achieved in clinical routine and in emergency settings without additional costs.

## 1. Introduction

Universal newborn hearing screening (UNHS) is crucial in the first month of life for facilitating early hearing detection and intervention (EHDI) in case of significant permanent hearing loss [1]. The goal of EHDI is the prompt management of deaf or hard of hearing (DHH) children with a view to minimizing hearing deprivation while maximally stimulating auditory development during the peak period of neural growth [2]. Since early auditory stimulation is the foundation of optimal speech and language development in the first year of life, EHDI facilitates linguistic competence and literacy development in DHH children [2].

In 2017, the Italian Ministry of Health introduced UNHS among the Essential Levels of Assistance [3]. In the Campania region, the third region in Italy and the first one in south Italy in terms of number of births, UNHS was introduced in 2003 by the Regional Council Resolution and it has been universally performed at three levels [4,5]. The first level consists of 56 birth centers in which there are about 55,000 births every year and of 18 neonatal intensive care units (NICU). The second level is composed of 15 corporate structures responsible for the confirmation of the diagnosis (departments of Audiology and Phoniatrics and of Otolaryngology). The third level is the Regional Reference Center (RRC), supervised by the Unit of Audiology and Vestibology of the Neuroscience Department of the University of Naples “Federico II”. The latter is responsible for the final treatment as well as the rehabilitation of children with hearing loss or deafness.

However, a diagnosis of permanent hearing impairment and a prescription of hearing aids are just the first step of a long habilitation program consisting in an intensive speech training with specialized pediatric therapists to promote the normal language development [6]. Indeed, the literature highlights the benefits of proper habilitation settings and points out that a family-centered approach seems to obtain better results because the family is the natural environment where a child grows up [7].

In order to start an appropriate EHDI program, an asynchronous model of telepractice has been developed by the Unit of Audiology and Vestibology of Federico II University Hospital in Naples and has been adopted as a tool for early intervention for DHH children since 2018. It is a web app for common devices called NeonaTal Assisted TelerehAbilitation (T.A.T.A.) for hearing-impaired children which allows monitoring the children’s learning skills to identify any possible specific difficulty.

It was implemented to follow more strictly both improvements and delayed development of DHH children and define the best habilitation strategies. Indeed, it appeared useful to encourage families who live far from qualified providers, who do not have the means to travel, or who may encounter challenges in accessing selected specialized early intervention services [1].

The aim of this article was to investigate parental self-efficacy beliefs and involvement and the language skills of DHH children who were habilitated with hearing aids and followed using the T.A.T.A. web app.

## 2. Materials and Methods

A prospective analysis was performed by the Unit of Audiology and Vestibology of the University of Naples Federico II between May 2019 and June 2021. The study describes the early stages of the habilitation program of 15 DHH children diagnosed within the UNHS program in Campania region and treated using the T.A.T.A. web app, an example of asynchronous model.

The T.A.T.A. app was designed and implemented by the Unit of Audiology and Vestibology of the University Hospital Federico II in Naples. It is available on common smart devices (smartphone, tablet) for interaction between a family and the hospital. It promotes familiar empowerment throughout a weekly questionnaire presented during the first 270 to 360 days of the baby’s life, for 14 weeks, focused on attentive audiological, communicative extraverbal and verbal, and neuromotor areas. The questionnaire contains 93 items and 7 to 10 questions per week. These concern the child’s behavior, in particular, his attention, auditory signal processing, oral praxis abilities, imitation and communication skills. The app is quite intuitive because simple scientific explanations are provided for each question to help evaluate the improvements in a specific ability. Moreover, it offers some suggestions for daily routines. Therefore, the use of the T.A.T.A. app encourages the observation of the child and the stimulation of his abilities to improve his communicative language and cognitive development. Counseling between the caregivers and the habilitative team starts a month after diagnosis to inform the families about aspects of language and communication development and to support DHH children by exposing them to language models at the earliest age in order to ensure their optimal cognitive, emotional, and educational development.

The families attend at least a monthly meeting until the infant’s age of 270 days. Indeed, their evaluation requires the use of family involvement rate scale (FIRS) to better determine the compliance of the parents to the program. Then, the child is enrolled, and the T.A.T.A. app is downloaded on the parental mobile. The protocol includes in-person visits at the beginning (first control point) and at the end of the training period (second control point) and the use of tools such as Infant Listening Progress Profile (ILiP), Category of Auditory Perception (CAP), MacArthur–Bates Communicative Development Inventories words and gesture (CDIs), TAIT videoanalysis, and Griffiths mental developmental scales (GMDS-ER). Moreover, at the first control point, the caregiver completes a baseline questionnaire that contains all items included in the app describing the skills already acquired by the child or emerging before the habilitation program. Then, the T.A.T.A. app can be used, and the family is followed through a web interface. Here, each patient’s neonatal history and diagnostic details are examined. It is always possible to read the stored information concerning neonatal clinical details, familiar anamnesis of audiological and neurodevelopmental disorders, results of UNHS and hearing tests (TEOAEs, DPOAEs, aABR, air and bone threshold ABR, tympanometry), and weekly questionnaire answers. Then, the parents are suggested to take a weekly semi-structured video of the baby during his activities, because videoanalysis will help to observe the child’s progress and to verify the answers of the parents. Furthermore, the family is always allowed to consult with the clinicians, and an alerting system sends a warning to them if something needs to be repeated or corrected. At the end of the 14th week, the evolutionary development profile is achieved: all questions are summarized in an endline questionnaire. Audiological, communicative–verbal, and neuropsychological evaluations are repeated to determine the benefits gained by the child and the limits of the T.A.T.A. treatment.

### 2.1. FIRS

The Family Involvement Rate Scale [8] (FIRS) permits the characterization of the quality of family participation in the intervention program through a global evaluation from 1 to 5 (1, limited participation; 2, below average participation; 3, average participation; 4, good; 5, ideal participation). At least 2 members of the medical team who interact with the family judged independently the levels of parental participation, and their results were compared. Complete agreement was found when both raters assigned the same point score. Categorical agreement was found when raters accurately placed families into 1 of 3 categories (e.g., 1–2, below average; 3, average; 4–5, above average), that is, the raters agreed on the category of assignment (which did not deviate by 2 or more points).

### 2.2. ILiP

The Infant Listening Progress Profile [9] (ILiP) is devised to monitor changes in the early auditory performance. The profile covers a range of abilities from first response to environmental sounds, through discrimination of environmental sounds and discrimination of voices, to identification of own name. It comprises 21 items. Children are scored according to whether their behavior is demonstrated always (2), sometimes (1), or never (0). The total score is the sum of the 21 scores. If there is any uncertainty in their response, ‘not known’ is recorded, and a score of zero is given. The maximum score is thus 42.

### 2.3. CAP

The Categories of Auditory Performance [10] (CAP) is an index used to measure the speech perception performance. It measures the supraliminal performance, reflecting everyday auditory performance in a more realistic way. It consists of eight performance categories arranged in order of increasing difficulty and comprising a hierarchical scale of auditory perceptive abilities ranging from 0, i.e., “displays no awareness of environmental sounds”, to 7, i.e., “can use the telephone with a familiar talker”.

### 2.4. Statistical Analysis

Data are presented as numbers or percentages for categorical variables. Continuous data are expressed as mean ± standard deviation (SD) or median with interquartile range (IQR).

The test used for normal distribution was the Shapiro–Wilk test.

The chi-square test and Fisher’s exact test were performed to evaluate significant differences of proportions or percentages between two independent samples. Particularly, the Fisher’s exact test was used where the chi-square test was not appropriate.

The *t*-test was used to test differences between two means of paired/unpaired data. Alternative non-parametric tests were used when the distribution was not normal. Particularly, the Mann–Whitney test was used to compare two independent samples, while the Wilcoxon test was used to compare two dependent samples.

The degree of association for each control point between Firs score and other parameters such as ILiP and CAP score, age, and gender was calculated using Spearman correlation coefficient.

Finally, to stratify the sample according to the FIRS scale, we considered the median value among possible scores (i.e., a score of 3)

All tests with *p*-value (*p*) < 0.05 were considered significant. The statistical analysis was performed by Matlab statistical toolbox version 2008 (MathWorks, Natick, MA, USA) for Windows at 32 bit.

## 3. Results

The sample included 15 consecutive DHH children including 73.33% of males and 26.67% of females, aged between 240 and 300 days, with a mean age of 258 days and a standard deviation of 18.97 days at the first control point (Pretest), and aged between 360 and 450 days, with a mean age of 410 days and a standard deviation of 46.29 days at the second control point (Posttest). The evaluation of the auditory perceptual skills and the family involvement were performed at these two control points. Table 1 reports the patients’ demographic characteristics.

At the first evaluation, the auditory–perceptual skills showed a mean value of 47.07 ± 27.11 for ILiP and a mean value of 1.93 ± 0.96 for CAP. Moreover, the mean value of FIRS was 2.80 ± 0.77. At the second control, the mean values were 67.53 ± 28.47 for ILiP, 3.13 ± 1.41 for CAP, and 3.87 ± 1.06 for FIRS.

To evaluate the family involvement in relation to other parameters, we defined subgroups considering the FIRS scale, i.e., Group A (posttest FIRS ≤ 3) and Group B (posttest FIRS > 3), as shown in Table 2. In this case, we considered the median score of the FIRS scale as a cut-off to define the FIRS groups. Particularly, this analysis was not performed at the first control point, because the Groups A and B were respectively composed of 3 and 12 children, i.e., they were very unbalanced.

We found a significant difference for the ILiP score, particularly at the second control point, when the children showed greater auditory–perceptual skills than at the first control point (67.53 vs. 47.06, *p* = 0.0030). This was observed also for the auditory performance scale (CAP, 3 vs. 2, *p* = 0.0093) and the FIRS scale (4 vs. 3, *p* = 0.0002). In other words, the auditory skills improved with older age as well as with increased parental participation.

When comparing Group A and Group B, we observed a significant difference for the auditory performance scale only (CAP, 2.86 vs. 3.38, *p* = 0.029).

Finally, we performed a univariate analysis of the relationship between the FIRS scale and other parameters such as age, gender, ILiP, and CAP scale for each control point. Since the distribution of the variables was not normal, the degree of association between the variables was calculated using the Spearman’s correlation coefficient rho, determining the *p*-value (Table 3).

As shown in Table 3, we observed a significant negative correlation between the FIRS scale and gender at the second control point only (rho = −0.52, *p* = 0.0456). In other words, a greater family involvement was associated with the female gender for children aged between 360 and 450 days.

## 4. Discussion

The use of hearing aids by DHH infants is just the first critical step to facilitate a timely and optimal maturation of the auditory system as a precursor to language development.

An extensive literature highlights the variables influencing the outcomes of hearing-impaired children and describes a strong positive relationship between family involvement and language development in the child. A study demonstrated that delivery models facilitating familiar inclusion are essential to improve a child’s language outcomes [8].

Moreover, speech therapy in an unfamiliar context is time-limited and places the child outside of his usual environment. The aim is to guarantee language development in time through a steady stimulation in normal life conditions. A significant correlation between the amount of early conversation between adults and child conversation and the growth of the child’s brain structure has been proved, specifically in terms of connectivity strength in the language tracts (dorsal white matter of the left hemisphere) [11,12] An early structural reorganization depends on neural commitment to language, and a significant correlation was found between the quantity of language input and speech perception at 11 to 14 months of age [12,13]. An early treatment was successively correlated with stronger vocabulary and verbal reasoning skills at 18 months [14] and 5 years of age [8]. Therefore, the family-centered system obtains the best improvements because it allows a continuous training of the child in the routine environment.

Several clinical investigations have shown how the feeling of self-efficacy in parents during the cure and management of a DHH child could have a positive effect on language development [15,16]. Therapists should teach caregivers (parents) how to interact with the affected child and to adopt new strategies for improving the child’s learning abilities. Nevertheless, variable results are obtained due to differences in the sociocultural familiar contexts. It is supposed that parental education positively influences the child’s development, because educated parents could better stimulate the child. Conversely, contrasting results are emerging regarding the family socio-economic status [17]. It was hypothesized that wealthy families can better help their children, because they may have access to more resources that support the development of the communicative skills of their child [8]. On the other hand, greater familiar economic possibilities are not always correlated with increased attention to the child when compared with unwealthy environments. Important factors are the quantity and the quality of the time spent with the child. Furthermore, maternal communicative skills seems to be an important aspect of parental involvement, because a parent must be thoroughly involved to develop an effective mutual communication with a DHH child [8]. In fact, a limited parental interaction causes a psychomotor delay that can lead to attentive and motor ipo- or iperactivation. The baby can have difficulties in acquiring attentive abilities, language development, and learning skills. For these reasons, a parental empowerment program has evolved as an asynchronous telepractice.

The T.A.T.A. web app supports the habilitation program in a very early period of life. To the best of our knowledge, other similar web applications are not described in the literature. Further, the monitoring of weekly outcomes permits the promotion of a tailored habilitation when the expected goals are not reached and eventually the adoption of new strategies to achieve the best results. It is also possible to check potential emerging problems which require prompt modifications of the habilitation program, as well as to follow several patients in the same period, providing more benefits at a low cost.

Nevertheless, this application does not replace in-person home visits or telecounseling rehabilitation programs, because it only consists of a questionnaire with the aim to better focus on a child’s abilities and to improve them. Its usefulness consists in its allowing therapists to follow young patients at home during a critical period for language development and to empower parents to promote their child’s skills. For this reason, the T.A.T.A. web app should not be compared to other programs such as LENA (Language Environment Analysis System), a device recording the real-word language exposure of a child, which has demonstrated that an early language experience may predict developmental outcomes years later [18].

These preliminary results encourage us to increase our sample of DHH children to better understand advantages and limits of this application and support future studies on evaluating language development and perceptual and curricular skills in DHH children who were habilitated with hearing aids and followed using the T.A.T.A web app. This tool has appeared to be a valid support because it has helped families to remain in contact with doctors and therapists. In fact, the T.A.T.A. app allows families not only to receive answers to questions related to the observation of their child’s behavior but also to send videos that illustrate particular situations or messages to request health support. Furthermore, the family is always allowed to chat with clinicians, and an alerting system sends a warning to them if something needs to be repeated or corrected. In detail, parents usually answered in time and usually talked with speech therapist at least once a week.

The health professionals, in turn, thanks to the web platform of the T.A.T.A. app, could check the status of each individual patient by offering the families suggestions for improving their child’s habilitation.

The use of telepractice adheres to the major tenets of early intervention services delivered in the natural environment of the child or in community settings where typical developing peers are found. Services can be family-centered and include parent coaching, direct or consultative, and support a range of multidisciplinary, interdisciplinary, or transdisciplinary teaming models. The use of telepractice in this field has grown significantly over the last 20 years. Recent studies demonstrate the effectiveness of teleintervention as a service delivery model [19,20,21], and suggest that telepractice could only supplement, but not replace, in-person sessions. Overall, the potential benefits of telepractice have been universally acknowledged, including its potential for improving the quality of services, increasing access to services, and enhancing the level of family centeredness.

## 5. Conclusions

In order to reap the benefits of familiar involvement, innovative services such as delivery models should be explored. The T.A.T.A. web app promotes a proactive management of DHH children through an active parental involvement. It provides a general and timely development profile of the child regarding emerging skills and critical points. Future developments will focus on realizing a more dynamic program to better tailor the telepractice and ameliorate the auditory stimulation in the absence of hearing impairments in patients with neurodevelopmental disorders.

## 6. Limitations

The current study has some limitations. First, the size of the sample for the analysis was relatively small in relation to the low prevalence of congenital hearing loss. On the other hand, the T.A.T.A web app has started to be increasingly adopted in early interventions for DHH children only recently.

Moreover, this study lacked a codified control group, but the use of this app seems to provide new opportunities to better follow the children and their families. In our experience, the improvement of auditory skills is not simply age-related. This is the first work about the application of the T.A.T.A. app, but another research has demonstrated that delivery models facilitating familiar inclusion are essential to improve children’s language outcomes [8].

For these reasons, the data presented should be interpreted as preliminary results, although they encourage multi-center studies with larger samples and similar demographic compositions. Despite the above-mentioned limitations, this study demonstrated that the clinical management of DHH children should include a family-centered system to achieve the greatest improvements, allowing children’s continuous training in their routine environments.

## Figures and Tables

**Table 1:** Characteristics of the 15 children monitored at two consecutive control points.

	Pretest	Posttest	Pretest vs. Posttest
Parameters	(240–300 Days)	(360–450 Days)	*p*-Value (Test Type)
Children	15	15	
Age (days)			
Mean ± SD	258 ± 18.97	410 ± 46.29	*p* < 0.0001 * (T)
Median (IQR)	270 (240–270)	390 (375–450)	
Gender			
Male	73.33% (11)	73.33% (11)	—
Female	26.67% (4)	26.67% (4)	
Auditory–perceptual skills			
ILiP (%)			
Mean ± SD	47.07 ± 27.11	67.53 ± 28.47	*p* = 0.0030 * (T)
Median (IQR)	50 (28–63)	75 (59.5–88)	
(CAP)			
Mean ± SD	1.93 ± 0.96	3.13 ± 1.41	
Median (IQR)	2 (1.5–2)	3 (2.5–4)	*p* = 0.0093 * (W)
Family participation in the intervention program			
Family involvement rate scale (FIRS)			
Mean ± SD;	2.80 ± 0.77	3.87 ± 1.06	
Median (IQR)	3 (2–3)	4 (3–5)	*p* = 0.0002 * (W)

ILiP = infant listening skill profile %; CAP = Categories of Auditory Performance. SD = standard deviation, IQR= interquartile range; *p* = *p*-value; * = significant statistical test; T = *t*-test; W = Wilcoxon test.

**Table 2:** Characteristics of the 15 children stratified according to a cut-off defined using the FIRS scale.

Parameters	Group A	Group B	Group A vs. Group B
	FIRS ≤ 3	FIRS > 3	*p*-Value (Test Type)
Children	7	8	
Age (days)			
Mean ± SD	415.71 ± 47.21	405 ± 48.11	*p* = 0.98 (T)
Median (IQR)	390 (390–450)	390 (360–450)	
Gender			
Male	100% (7)	50.0% (4)	*p* = 0.077 (F)
Female	0.0% (0)	50.0% (4)	
Auditory–perceptual skills			
ILiP (%)			
Mean ± SD	67.0 ± 10.66	68 ± 39.02	
Median (IQR)	75 (59.5–75)	88 (59.25–89.25)	*p* = 0.16 (MW)
CAP			
Mean ± SD	2.86 ± 0.69	3.38 ± 1.85	*p* = 0.029 * (T)
Median (IQR)	3 (2.5–3)	4 (3.25–4.25)	
Family participation in the intervention program			
Family involvement rate scale (FIRS)			
Mean ± SD	2.86 ± 0.38	4.75 ± 0.46	
Median (IQR)	3 (3–3)	5 (4.75–5)	*p* = 0.0005 * (MW)

ILiP = infant listening skill profile %; CAP = Categories of Auditory Performance; SD = standard deviation, IQR = Interquartile range. *p* = *p*-value; * = significant statistical test; T = *t*-test; MW = Mann-Whitney test; F = Fisher’s exact test.

**Table 3:** Correlation analysis of the relationship between the Family Involvement Rate Scale and other parameters such as age, gender, ILiP, and CAP scale.

Parameters	Correlation Test Rho (*p*-Value)
First Control Point	
Firs/Age	−0.17 (0.55)
Firs/Gender	−0.39 (0.15)
Firs/ILiP	−0.012 (0.97)
Firs/CAP	−0.08 (0.79)
Second Control Point	
Firs/Age	−0.27 (0.33)
Firs/Gender	−0.52 (0.0456) *
Firs/ILiP	0.38 (0.17)
Firs/CAP	0.39 (0.15)

rho = Spearman’s coefficient; * significant test (*p* < 0.05); *p* = *p*-value.

## Data Availability

The data will be available on the website of the Audiology unit www.audiologia.unina.it (accessed on 1 March 2022) after the publication of the article.
